# Depletion of macrophages and osteoclast precursors mitigates iron overload‐mediated bone loss

**DOI:** 10.1002/iub.2928

**Published:** 2024-11-18

**Authors:** Vanessa Passin, Maria G. Ledesma‐Colunga, Sandro Altamura, Martina U. Muckenthaler, Ulrike Baschant, Lorenz C. Hofbauer, Martina Rauner

**Affiliations:** ^1^ Department of Medicine III & Center for Healthy Aging Medical Faculty and University Hospital Carl Gustav Carus, Dresden University of Technology Dresden Germany; ^2^ Department of Pediatric Hematology, Oncology and Immunology University of Heidelberg Heidelberg Germany; ^3^ Molecular Medicine Partnership Unit European Molecular Biology Laboratory Heidelberg Germany

**Keywords:** bone loss, C57BL/6J, clodronate, iron overload, osteoclast

## Abstract

Iron is an essential element for physiological cellular processes, but is toxic in excess. Iron overload diseases are commonly associated with low bone mass. Increased bone resorption by osteoclasts as well as decreased bone formation by osteoblasts have been implicated in bone loss under iron overload conditions. However, the exact contribution of individual cell types has not yet been formally tested. In this study, we aimed to investigate the role of osteoclast precursors in iron overload‐induced bone loss. To that end, we used clodronate liposomes to deplete phagocytic cells (including macrophages and osteoclast precursors) in male C57BL/6J mice that were exposed to ferric derisomaltose. Bone microarchitecture and bone turnover were assessed after 4 weeks. The application of clodronate resulted in the efficient depletion of circulating myeloid‐lineage cells by about 70%. Depletion of osteoclast precursors mitigated iron overload‐induced trabecular bone loss at the lumbar vertebrae and distal femur. While clodronate treatment led to a profound inhibition of bone turnover in control mice, it significantly reduced osteoclast numbers in iron‐treated mice without further impacting the bone formation rate or serum PINP levels. Our observations suggest that even though bone formation is markedly suppressed by iron overload, osteoclasts also play a key role in iron overload‐induced bone loss and highlight them as potential therapeutic targets.

## 1 INTRODUCTION

Iron is an essential element of life participating in various physiological processes. Iron in excess however is toxic. Therefore, tightly balanced iron levels are essential for health. Amongst many organs affected by iron excess, bone is highly susceptible to imbalanced iron levels. Osteoporosis and an increased fracture prevalence commonly occur in iron overload diseases such as hereditary hemochromatosis, β‐thalassemia, and sickle cell disease.^1^, ^2^, ^3^ These clinical observations are supported by experimental animal studies investigating the impact of exogenous iron administration or genetic iron overload on bone microarchitecture.^4^ Bone remodeling is critical to ensure healthy bones and relies on the tightly balanced processes of bone resorption and bone formation. Growing evidence suggests that iron overload disrupts bone homeostasis due to both increased bone resorption by osteoclasts and decreased bone formation by osteoblasts. However, it is still debated what cell type may be the main driver of the underlying bone loss.

Osteoblasts derive from mesenchymal stem cells and facilitate the formation and mineralization of new bone matrix. Iron overload impairs the proliferation, differentiation, and mineralization capacity of osteoblasts.^5^, ^6^, ^7^ In vivo studies also suggest decreased bone formation in various conditions of iron overload disease models.^8^, ^9^, ^10^ Osteoclasts are multinucleated cells that resorb damaged bone by releasing protons and proteolytic enzymes that facilitate the degradation of the bone matrix. They originate from the monocyte/macrophage lineage. Macrophages play a crucial role in iron recycling by degrading erythrocytes.^11^ Macrophages require iron to maintain their metabolic demands and proper immune responses,^12^, ^13^ as do osteoclasts for mitochondrial biogenesis and proper bone‐resorbing function.^14^ In vitro studies suggest that excess iron accelerates not only the differentiation of osteoclasts but also their activity and subsequently bone resorption.^14^, ^15^, ^16^ In vivo studies in iron‐overloaded C57BL/6 mice and mouse models of hemochromatosis observed an increased number of osteoclasts and increased serum levels of the bone resorption marker C‐telopeptide of type‐I collagen.^17^, ^18^, ^19^ These studies suggest that increased osteoclast activity could play a central role in iron overload‐associated bone loss.

In this study, we aimed to investigate the significance of osteoclasts in iron overload‐associated bone loss. To address this, we depleted phagocytic cells including macrophages and monocytes as precursors of osteoclasts in the circulation using clodronate liposomes in male iron‐overloaded C57BL/6J mice. We found that depletion of monocyte‐derived osteoclasts mitigated the effect of iron overload on bone. Our results suggest that osteoclasts account for iron overload‐induced bone loss and thus, add to a better understanding of the underlying mechanisms and potential therapeutic options for iron‐related bone disease.

## 2 EXPERIMENTAL PROCEDURES

### 2.1 Mice

All animal procedures were conducted in compliance with the faculty guidelines on Animal Welfare and were approved by Landesdirektion Sachsen, Germany (TVV 20/2020). Eleven‐week‐old male C57BL/6J mice were purchased from Janvier Labs (France) and maintained in groups of four animals per cage under a 12 h dark/light cycle at controlled temperature (23°C) with food (standard chow containing 176 mg iron/kg, Ssniff V1534‐3) and water ad libitum.

**FIGURE 1:**
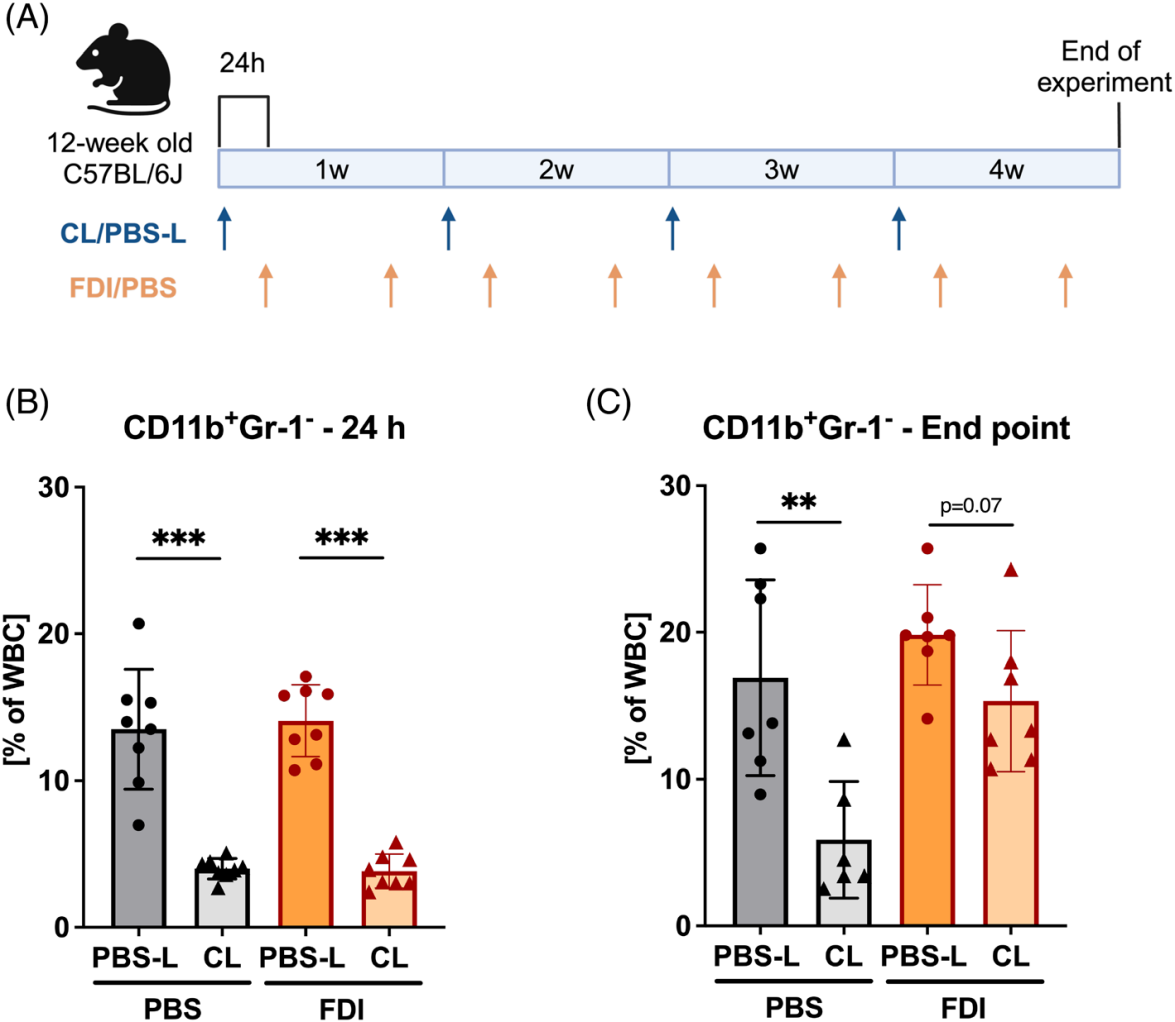
Experimental treatment scheme. Twelve‐week‐old male C57BL/6J mice received clodronate liposomes (CL) i.v. once a week for 4 weeks. Ferric derisomaltose (FDI) was injected i.p. twice a week for 4 weeks, the first FDI injection of each week was administered 24 h after the CL injection (A). CD11b^+^Gr1^−^ cell depletion was confirmed 24 h after the first CL injection (before first FDI injection) (B) and at the end of the experiment after 4 weeks (C). Data are represented as mean ± SD (*n* = 8 per group). Cell populations are presented as % of white blood cells (WBC). Each symbol represents an individual animal. Statistics were calculated using Student's *t*‐test. **p < .01, ***p < .001.

At 12 weeks of age, iron overload in mice was induced by administration of ferric derisomaltose (FDI; MonoFer, Pharmacosmos) via intraperitoneal (i.p.) injections twice a week over 4 weeks at a dose of 500 μg/g of body weight. The dose was determined based on previous studies.^19^, ^20^, ^21^ Concomitantly, clodronate liposomes (CL, Liposoma, #CP‐005‐005) were injected intravenously (i.v., 25 μg/g of body weight) for 4 weeks once every week (Figure 1A). As a control, phosphate‐buffered saline liposomes (PBS‐L) were used. The mice were randomly divided into four groups with eight animals per group: (i) a control group with PBS and PBS‐L, (ii) a control group with PBS and CL, (iii) an iron‐overloaded group with FDI and PBS‐L, (iv) an iron‐overloaded group with FDI and CL. At the end of the experiment, mice were euthanized with CO_2_ and their tissues were collected for further analysis.

### 2.2 Flow cytometry

Peripheral blood was obtained from the retro‐orbital plexus of mice under isoflurane anesthesia and collected in PBS supplemented with 5 mM EDTA. After centrifugation, the cell pellet was resuspended in ACK lysis buffer (Gibco) for 2 min. This step was repeated one more time before staining the cells for 30 min using antibodies specific for the following antigens: CD11b‐APC (eBioscience, #17‐0112‐81), Gr‐1(Ly‐6C/G)‐FITC (BioLegend, #108406). Samples were acquired using the LSR Fortessa Cell Analyzer (BD) and the results analyzed using FlowJo™ v10.8 software (BD). The gating strategy is displayed in Figure S1. Cell populations are presented as percentage of white blood cells (WBC).

### 2.3 Blood and serum measurements

Blood obtained from heart puncture was analyzed using an automated cell counter (Sysmex). For serum collection, blood was further processed by centrifugation for 20 min at 3000 rpm. Serum iron concentration was determined using the IRON (SFBC) Bathophenanthrolin kit (Biolabo) and unsaturated iron‐binding capacity (UIBC) was measured with the UIBC kit (Biolabo). Transferrin saturation was calculated with the formula: (SFBC/[SFBC + UIBC]) x 100. Tartrate‐resistant acid phosphatase form 5b (TRAcP5b) and N‐terminal propeptide of type I procollagen (PINP) as markers of bone turnover were measured in the serum with Enzyme‐linked Immunosorbent Assays (Immunodiagnostic Systems, UK) according to the manufacturer's protocol.

### 2.4 Determination of tissue iron content

Small pieces of liver and spleen were collected, dried at 37°C for 72 h and incubated in 10% trichloroacetic acid/10% hydrochloric acid in distilled water for 48 h at 65°C and 300 rpm. Non‐heme iron content was determined using the bathophenanthroline colorimetric method.^22^ Iron content is reported as μg of iron per g of dry tissue weight calculated based on serial dilution of a ferric iron standard (Sigma‐Aldrich).

### 2.5 Micro‐computer tomography

Bone microarchitecture from the excised femur and L4‐vertebral body was assessed using the micro‐CT vivaCT 40 (Scanco Medical) with an isotropic voxel size of 10.5 μm (70 kVp, 114 μA, and 200 ms integration time). The trabecular bone compartment at the distal femur and mid‐vertebrae was isolated by manual contouring, each compartment included regions spanning 100 slices. For analysis, established protocols from Scanco Medical were applied to assess trabecular parameters such as bone volume per total volume (BV/TV) and trabecular thickness (Tb.Th). Analysis of cortical bone comprised analysis of the cortical thickness (Ct.Th) at the femoral midshaft (using 150 slices). The results are reported following international guidelines.^23^

### 2.6 Bone histomorphometry

To assess the number of osteoclasts per bone perimeter (N.Oc/B.Pm) and number of osteoblasts per bone perimeter (N.Ob/B.Pm), TRAP staining was performed on 2 μm paraffin sections of decalcified vertebral bodies in an area of 1.44 mm^2^. To assess bone formation, mice received i.p. injections of calcein (20 mg/kg, Sigma) on day 5 and 2 before sacrifice. Dissected bones were fixated in 4% paraformaldehyde for 48 h and dehydrated in an ascending ethanol series. The third to fourth lumbar vertebral body was embedded in methyl methacrylate (Technovit). Fluorescence labeling was assessed on 7 μm sections. The mineral surface per bone surface (MS/BS), and mineral apposition rate (MAR) were measured in an area of 1.44 mm^2^. All measurements were performed using the Osteomeasure software (OsteoMetrics).

### 2.7 Statistics

Student's unpaired two‐tailed *t*‐test was performed to calculate statistical differences between the PBS‐L and CL groups. A two‐way ANOVA (post‐hoc Tukey) was performed to compare multiple groups. The Grubb's test (*α* = 0.1) was employed to detect and remove outliers. All statistical analyses were performed using GraphPad Prism 10.0 (GraphPad Software Inc.). The data is represented as mean ± standard deviation (SD). Individual mouse data are shown as dots. Significance levels were denoted as values of *p < .05, **p < .01, or ***p < .001.

## 3 RESULTS

### 3.1 Clodronate liposome treatment efficiently depletes circulating CD11b ^+^Gr‐1^−^ cells

To study the role of osteoclasts on iron overload‐induced bone loss, we utilized clodronate liposomes (CL) to deplete phagocytic cells, including monocytes as the precursors of osteoclasts, in male C57BL/6J mice treated with either ferric derisomaltose (FDI) or PBS. PBS liposomes (PBS‐L) were used as a control. The depletion of circulating myeloid CD11b^+^Gr‐1^−^ cells was confirmed using flow cytometry (Figure S1) 24 h after the first CL injection as well as at the end of the experiment (Figure 1A). Twenty‐four hours after liposome injection, circulating CD11b^+^Gr‐1^−^ cell levels were significantly reduced from 14% to 4% of WBC (Figure 1B). At the end of the experiment, circulating myeloid cells were quantified again, 5 days after the final liposome injection. At that time point, CD11b^+^Gr‐1^−^ cells were reduced from 17% to 6% of WBC in the PBS‐treated group, whereas in iron‐treated mice, myeloid cells were only reduced from 20% to 15% of WBC (Figure 1C). Thus, overall, the application of CL led to a marked reduction of circulating myeloid CD11b^+^Gr‐1^−^ cells, which also include monocytes, the known precursors for osteoclasts.^24^

### 3.2 Clodronate liposome treatment does not change blood and iron parameters in iron‐overloaded mice

Next, we examined the impact of phagocyte depletion on hematological and iron parameters (Table 1 and Figure S2) CL treatment in the PBS‐treated mice resulted in reduced counts of reticulocytes as well as decreased levels of hemoglobin, hematocrit, and a slight reduction in transferrin saturation. Liver and serum iron levels were comparable to control mice, but splenic iron content was mildly decreased in CL‐treated mice (Table 1). FDI treatment markedly increased serum iron levels, transferrin saturation as well as the iron content of the liver and spleen whereas red blood cell count, and hematocrit levels were significantly reduced. Under iron‐overloaded conditions, CL‐treated mice also showed a significant reduction in splenic iron in comparison to the PBS‐L control mice (Table 1). We also observed increased levels of white blood cells in both groups of FDI‐treated mice. Intriguingly, CL treatment in iron‐overloaded mice significantly increased the number of circulating monocytes. Neutrophil counts were increased in FDI‐treated mice, but were unaltered in response to CL treatment. Contrarily, CL treatment reduced the elevated lymphocyte numbers in FDI‐treated mice (Table 1). Overall, CL treatment did not markedly change the iron profile of iron‐treated mice.

**TABLE 1:** Blood and iron parameters of control (PBS‐L) and clodronate‐treated (CL) C57BL/6J mice after receiving injections of ferric derisomaltose (FDI) for 4 weeks.

	PBS			FDI		
	PBS‐L (*n* = 7)	CL (n = 6)	*p*	PBS‐L (*n* = 6)	CL (*n* = 6)	*p*
Body weight [g]	27.3 ± 1.8	26.5 ± 0.9	.78	25.4 ± 1.9	25.0 ± 0.9	.99
Red blood cells [10^12^/L]	8.9 ± 0.5	8.2 ± 0.3	.13	7.7 ± 0.4^##^	8.0 ± 0.5^#^	.90
Hemoglobin [mmol/L]	8.1 ± 0.4	6.2 ± 1.3	**.004**	7.5 ± 0.3	7.6 ± 0.4	.99
Hematocrit [L/L]	0.47 ± 0.02	0.33 ± 0.06	**.000**	0.40 ± 0.01^#^	0.40 ± 0.03^#^	.99
Reticulocytes [10^9^/L]	660 ± 217	237 ± 150	**.009**	923 ± 206	571 ± 206	.06
White blood cells [10^9^/L]	9.1 ± 1.6	10.7 ± 2.2	.84	16.4 ± 1.4^##^	14.6 ± 4.6^#^	.79
Monocytes [10^9^/L]	0.09 ± 0.06	0.12 ± 0.04	.99	0.23 ± 0.09	0.63 ± 0.29^###^	**.005**
Neutrophils [10^9^/L]	0.28 ± 0.15	0.31 ± 0.09	.98	0.61 ± 0.24	0.77 ± 0.23^##^	.47
Lymphocytes [10^9^/L]	2.6 ± 0.5	3.0 ± 0.8	.84	4.6 ± 0.4^###^	2.9 ± 0.5	**.008**
Serum iron [μg/dL]	298 ± 18	284 ± 26	.99	1233 ± 333^###^	1566 ± 120^###^	.07
Transferrin saturation [%]	46.5 ± 2.2	37.7 ± 2.0	.09	88.7 ± 8.7^###^	87.4 ± 3.8^###^	.97
Liver iron content [μg/g of dry tissue]	171 ± 48	294 ± 147	.99	4034 ± 3429^#^	1680 ± 1703	.12
Spleen iron content [μg/g of dry tissue]	505 ± 161	249 ± 43	.90	10,355 ± 79^###^	8727 ± 984^###^	**.003**

*Note*: Significances within treatment groups are highlighted in bold. ^#^ Comparison to untreated control mice. Data are presented as mean ± SD. Statistics were calculated using two‐way ANOVA (post‐hoc Tukey). ^#^ *p* < .05, ^##^ *p* < .01, ^###^ *p* < .001.

### 3.3 Clodronate liposome treatment ameliorates iron overload‐induced bone loss

**FIGURE 2:**
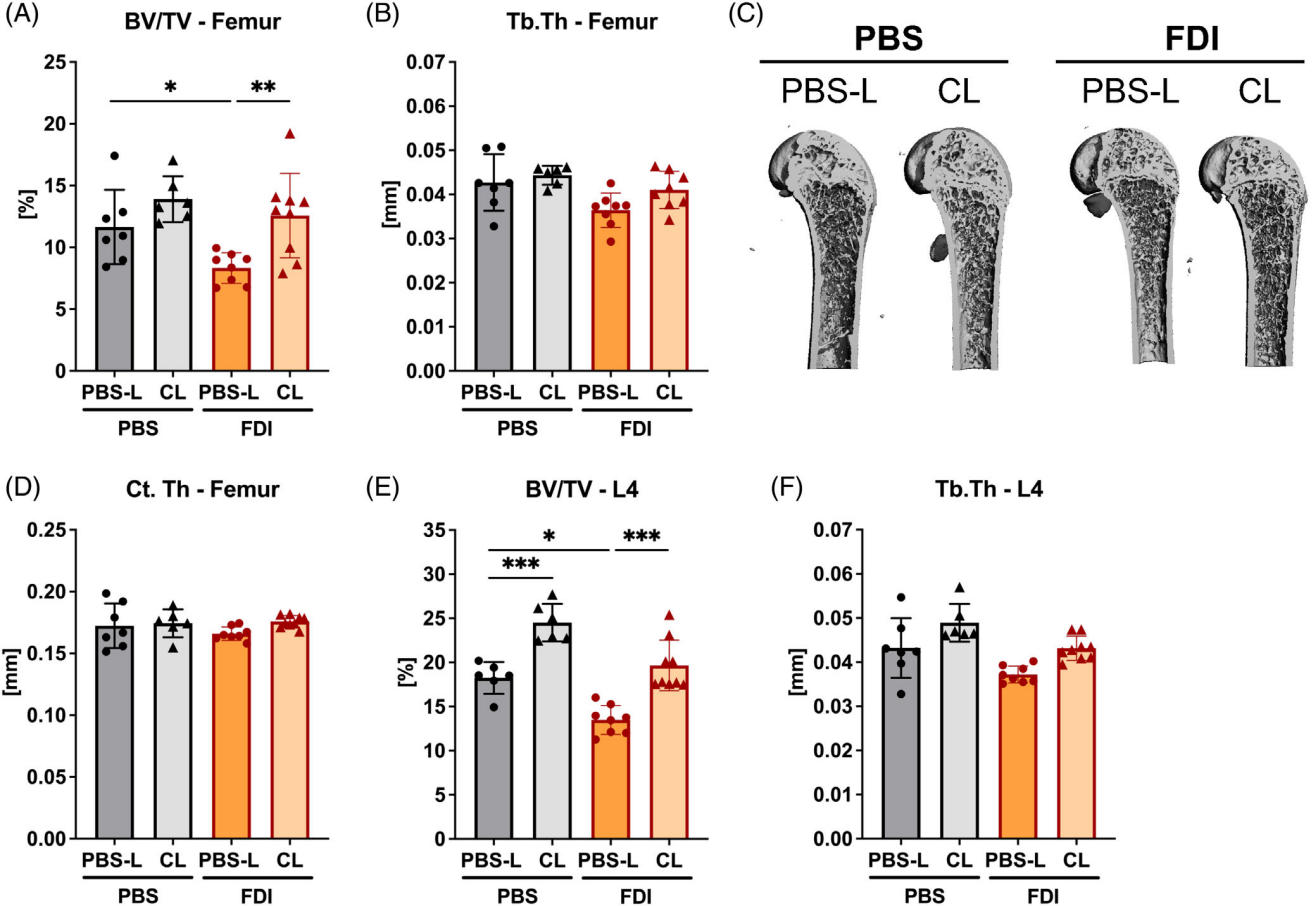
Clodronate liposome treatment mitigates iron overload‐induced bone loss. Bone parameters of control (PBS‐L) and clodronate‐treated (CL) C57BL/6J mice after receiving injections of ferric derisomaltose (FDI) for 4 weeks. Micro‐CT analysis was used to assess the trabecular bone parameters BV/TV (A,E) and Tb.Th (B,F) of the femur (A,B) and the fourth lumbar (L4) spine (E,F). Representative 3D images of trabecular bone from the femur are shown (C). Cortical thickness at the femoral midshaft was assessed (D). Data are represented as mean ± SD (*n* = 6–8 per group). Each symbol represents an individual animal. Statistics were calculated using a two‐way ANOVA (post‐hoc Tukey). *p < .05, **p < .01, ***p < .001.

Given that excess iron impacts on the differentiation and activity of osteoclasts and subsequently bone resorption, we assessed the bone microarchitecture of the femur and L4‐lumbar vertebrae using micro‐CT. FDI treatment led to a significant reduction of trabecular bone volume and a minor decrease in trabecular thickness in the femur, which could be reversed by CL treatment (Figure 2A–C). Cortical thickness remained unaffected by FDI and CL treatment (Figure 2D). Similarly, at the lumbar spine, FDI treatment led to a 15%–25% reduction of trabecular bone volume and thickness, which were reinstated to control levels following CL treatment (Figure 2E,F). Taken together, our observations suggest that monocyte depletion with CL mitigates bone loss under iron overload conditions.

### 3.4 Clodronate liposome treatment reduces osteoclast numbers in iron‐treated mice

**FIGURE 3:**
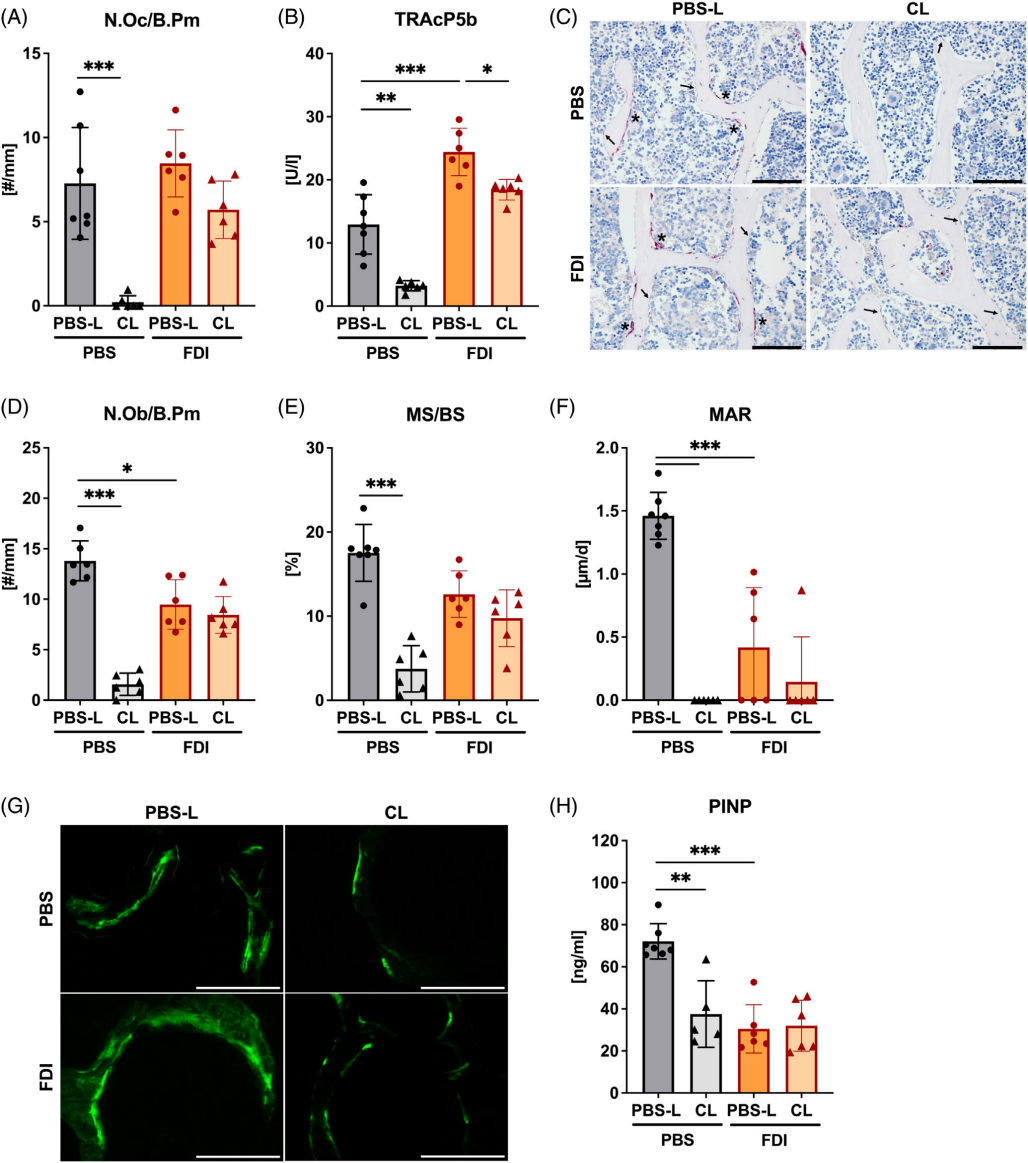
Clodronate liposome treatment reduces bone turnover. Bone resorption and bone formation markers of control (PBS‐L) and clodronate‐treated (CL) C57BL/6J mice after receiving injections of ferric derisomaltose (FDI) for 4 weeks. Bone resorption parameters (A–C) including TRAP staining of the L4 vertebral body to assess the number of osteoclasts (A). Serum levels of bone resorption marker TRAcP5b were measured (B). Representative images of TRAP staining are shown (C). Bone formation parameters (D–H) including TRAP staining of the L4 vertebral body to assess the number of osteoblasts (D) and calcein labeling of the L4 vertebral body was used to assess the mineral surface (E) and mineral apposition rate (F). Representative images of calcein labeling are shown (G). Serum levels of bone formation marker PINP were measured (H). Data are represented as mean ± SD (*n* = 6 per group). Each symbol represents an individual animal. Statistics were calculated using a two‐way ANOVA (post‐hoc Tukey). *p < .05, **p < .01, ***p < .001. Scale bar, 100 μm. Asterisks indicate areas with osteoclasts. Arrows indicate areas with osteoblasts.

To assess the influence of CL treatment on bone turnover, we performed static and dynamic histomorphometry of the lumbar spine. To further complement the histological evaluations, plasma levels of the bone resorption marker TRAcP5b and bone formation marker PINP were analyzed. As expected, the administration of CL to PBS‐treated control mice nearly eradicated the presence of osteoclasts (Figure 3A,C). Correspondingly, serum TRAcP5b levels were reduced by 80% following CL treatment in control mice (Figure 3B). While iron treatment did not induce an increase in the number of osteoclasts at histological level compared to control mice, TRAcP5b serum levels were increased (Figure 3A–C). CL treatment in FDI‐treated mice reduced the number of osteoclasts assessed by histology and levels of serum TRAcP5b to levels comparable to the PBS/PBS‐L control group (Figure 3A–C).

As bone formation and bone resorption are coupled processes, we next assessed the effect of monocyte depletion on osteoblasts. The number of osteoblasts as well as mineralizing surface and mineral apposition rate were reduced in the CL‐treated control mice (Figure 3D–F). Under CL treatment, calcein staining revealed missing double labels indicating a suppressed bone formation (Figure 3G). Along those lines, also the serum bone formation marker PINP was reduced by 50% after CL treatment in control mice (Figure 3H). FDI treatment reduced the number of osteoblasts and the histological parameters of bone formation, which were not further changed by CL treatment (Figure 3D–F). Moreover, serum levels of PINP were significantly reduced by iron and were not further altered by CL treatment (Figure 3H).

Taken together, this data suggests that monocyte depletion with CL reduces the iron‐induced activity of osteoclasts, but does not inhibit iron‐reduced bone formation further.

## 4 DISCUSSION

Numerous studies have shown the detrimental effect of iron overload on bone, proposing an increased osteoclast activity and decreased osteoblast activity as the underlying cause of pathological bone loss.^25^ In this study, we sought to examine the importance of osteoclasts and their precursors for bone loss in iron overload conditions. Therefore, phagocytic cells in C57BL/6J mice were depleted with CL and bone microarchitecture and turnover were evaluated.

As circulating monocytes have been identified as osteoclast precursors in adult mice,^24^ we aimed to delete this population to limit osteoclastogenesis, especially under iron overload conditions. Liposomes loaded with clodronate have been widely used to deplete phagocytic populations in mice.^26^, ^27^, ^28^, ^29^ We opted to use weekly injections of CL to achieve continuously low numbers of monocytes/macrophages as a previous study showed that monocyte numbers recover within 5 days of depletion.^28^ Indeed, our flow cytometric analysis showed markedly reduced numbers of circulating CD11b^+^Gr1^−^ cells after 24 h, as reported by other studies as well.^27^, ^28^ However, at the end of the experiment, circulating myeloid cell numbers were not as drastically reduced in the CL‐treated iron‐overloaded group, which may be explained by the 5‐day duration since the last CL injection before sacrifice. Furthermore, we found an increased number of monocytes in the circulation of iron‐overloaded CL‐treated mice which could have contributed to a faster population replenishment. It may therefore be envisaged that a more frequent application of CL, for example, every 2–3 days would have prevented macrophage repopulation and resulted in even more pronounced effects. However, osteoclast numbers in control mice were already reduced to almost zero showing that the treatment scheme was effective.

Interestingly, we observed significant changes in the iron‐related blood parameters of CL‐treated mice. Since macrophages play a crucial role in iron recycling from red blood cells,^11^ their depletion likely leads to reduced iron availability and hemoglobin synthesis.^30^ Serum iron levels do however not reflect this lack of iron recycling. Reduced macrophage numbers also account for the reduced spleen iron content in CL‐treated mice. Furthermore, several studies observed changed erythropoiesis in response to CL treatment, highlighting the role of macrophages in this process.^30^, ^31^, ^32^, ^33^ FDI administration resulted in iron accumulation in liver and spleen as well as increased serum iron levels, and introduced aberrant systemic iron metabolism, affecting hemoglobin and red blood cell production, potentially due to an exhaustion of erythropoiesis. Earlier studies did not observe changes in peripheral blood parameters in mouse models of iron overload but indicated an impaired bone marrow hematopoietic environment.^34^, ^35^ The increased number of white blood cells after FDI treatment may indicate an inflammatory response after acute iron administration. Neutrophil numbers however remained unaffected by the treatment with CL. Previous studies showed that CL uptake by neutrophils does not affect their numbers but alters their function.^36^ Future studies using disease models driven by neutrophils will be needed to address their relevance in this setting. Interestingly, CL treatment rescued the FDI‐mediated increase in lymphocyte numbers, suggesting that monocytes/macrophages may drive the increase in lymphocyte numbers after iron treatment. This finding is consistent with the proinflammatory response to iron accumulation in macrophages.^13^, ^37^

Iron overload induced by FDI administration significantly reduced bone mass and increased serum TRAcP5b levels, but did not change osteoclast numbers assessed via histology. This contrasts to previous studies that report increased osteoclast numbers in different conditions of iron overload.^17^, ^19^, ^38^ Our findings may indicate an increased osteoclast activity, as previously observed.^15^, ^18^ Iron overload also had a significant effect on osteoblast numbers, serum levels of PINP, and bone turnover. Iron overload is known to reduce osteoblast activity and reduce bone formation.^8^, ^9^, ^10^ CL treatment mitigated the bone loss induced by iron overload by decreasing osteoclast numbers and serum levels of TRAcP5b. CL treatment in control mice also resulted in decreased osteoblast numbers and PINP levels. As bone formation and bone resorption are tightly coupled, we suspect that not only bone resorption but also bone formation is slowed by CL administration. The effect of CL treatment parallels those of bisphosphonate treatment in osteoporosis, in which not only bone resorption but also bone formation is reduced subsequently slowing overall bone turnover due to coupling of both processes.^39^, ^40^ Since CL treatment also had an apparent effect on osteoblasts, our study does therefore not exclusively address the role of osteoclasts. Under iron overloaded conditions, CL did however not further reduce osteoblast numbers and PINP levels.

The process of bone homeostasis involves intricate interactions of multiple cell types. While our study highlights the role of osteoclasts in iron‐associated changes in bone homeostasis, recent studies have addressed different cell types. A study focusing on the role of osteocytes in hepcidin‐deficient mice suggests increased osteocyte apoptosis and increased sclerostin and RANKL/OPG as main reasons for reduced bone mass.^41^ A study focusing on the role of osteoblasts, reported that inhibition of ferroptosis in osteoblasts promoted osteogenesis in iron dextran‐treated mice.^42^ Further research is needed to get a clear picture about the contribution of the individual cell types to iron overload‐induced bone loss.

In conclusion, our study suggests that despite low bone formation, inhibition of osteoclasts is sufficient to limit iron‐induced bone loss highlighting anti‐resorptive therapies as useful therapeutic interventions to maintain bone mass under conditions of iron overload.

## FUNDING INFORMATION

This research was supported by grants from Deutsche Forschungsgemeinschaft (Ferros FOR‐5146) to UB, LCH, SA, MUM, and MR.

## CONFLICT OF INTEREST STATEMENT

MR reports honoraria for lectures and advisory boards from UCB and Vifor Pharma; LCH reports honoraria for advisory boards from Amgen, Ascendis, Pharmacosmos, and UCB to his institution and himself. MUM reports honoraria for lectures and advisory boards from Salus GmbH and Novonordisc. All other authors have nothing to disclose.

## Supporting information


**FIGURE S1.** Gating strategy to analyze CD11b^+^Gr1^−^ population. White blood cells (WBC) were gated in FSC‐SSC to exclude debris and then gated as singlets (FSC‐A/FSC‐H). From the living singlets CD11b^+^Gr1^−^ cells were gated as indicated.

**FIGURE S2.** Blood and iron parameters of control (PBS‐L) and clodronate‐treated (CL) C57BL/6Jmice after receiving injections of ferric derisomaltose (FDI) for 4 weeks. Data are represented as mean ± SD (*n* = 6–7 per group). Each symbol represents an individual animal. Statistics were calculated using a two‐way ANOVA (post‐hoc Tukey). *p < .05, **p < .01, ***p < .001.

## Data Availability

The data that support the findings of this study are available on request from the corresponding author (MR).
