# Supplementary figures and images for: Depletion of macrophages and osteoclast precursors mitigates iron overload‐mediated bone loss
Source: IUBMB Life. 2024 Nov 18;77(1):e2928. doi: 10.1002/iub.2928 (PMC11611226; doi:10.1002/iub.2928)

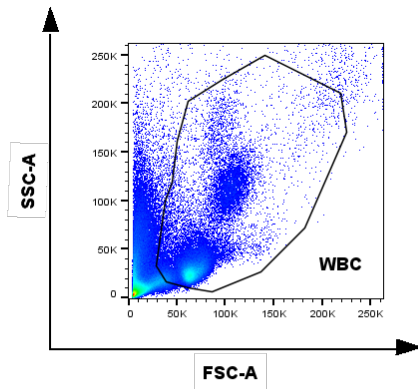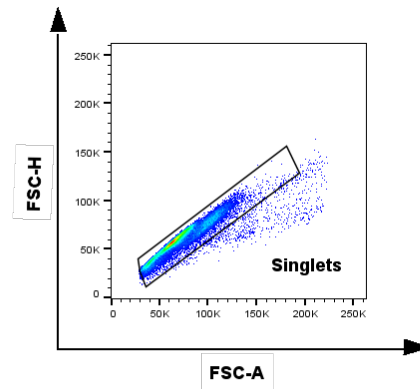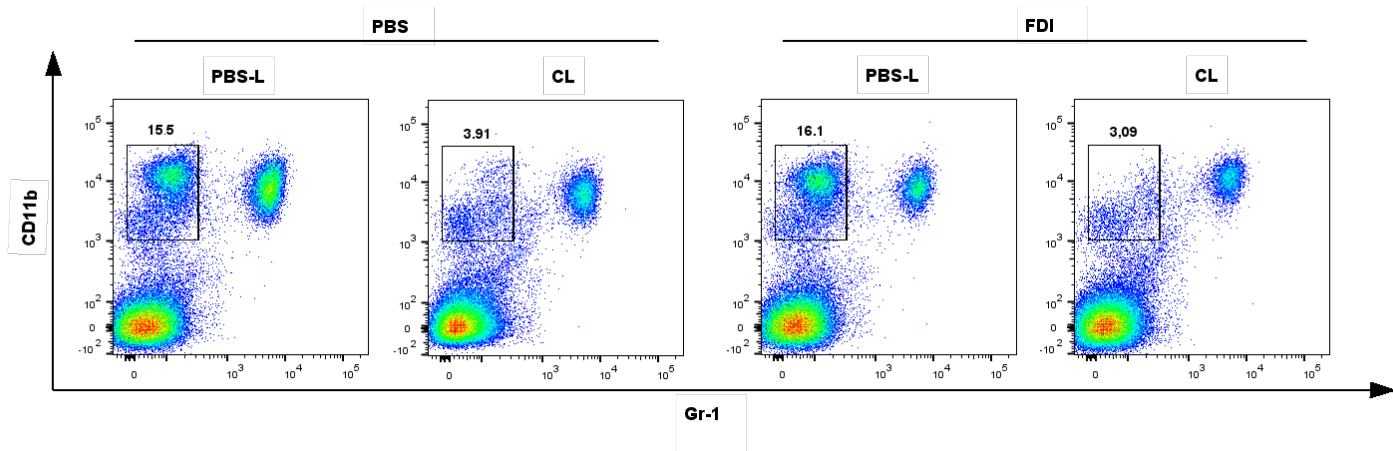

Supplement: Supplementary file 1 — FIGURE S1. Gating strategy to analyze CD11b+Gr1− population. White blood cells (WBC) were gated in FSC‐SSC to exclude debris and then gated as singlets (FSC‐A/FSC‐H). From the living singlets CD11b+Gr1− cells were gated as indicated. [file IUB-77-0-s002.pdf]
