# Supplementary material for: Depletion of macrophages and osteoclast precursors mitigates iron overload‐mediated bone loss
Source: IUBMB Life. 2024 Nov 18;77(1):e2928. doi: 10.1002/iub.2928 (PMC11611226; doi:10.1002/iub.2928)
Supplement: Supplementary file 2 — FIGURE S2. Blood and iron parameters of control (PBS‐L) and clodronate‐treated (CL) C57BL/6Jmice after receiving injections of ferric derisomaltose (FDI) for 4 weeks. Data are represented as mean ± SD (n = 6–7 per group). Each symbol represents an individual animal. Statistics were calculated using a two‐way ANOVA (post‐hoc Tukey). *p < .05, **p < .01, ***p < .001. [file IUB-77-0-s001.pdf]

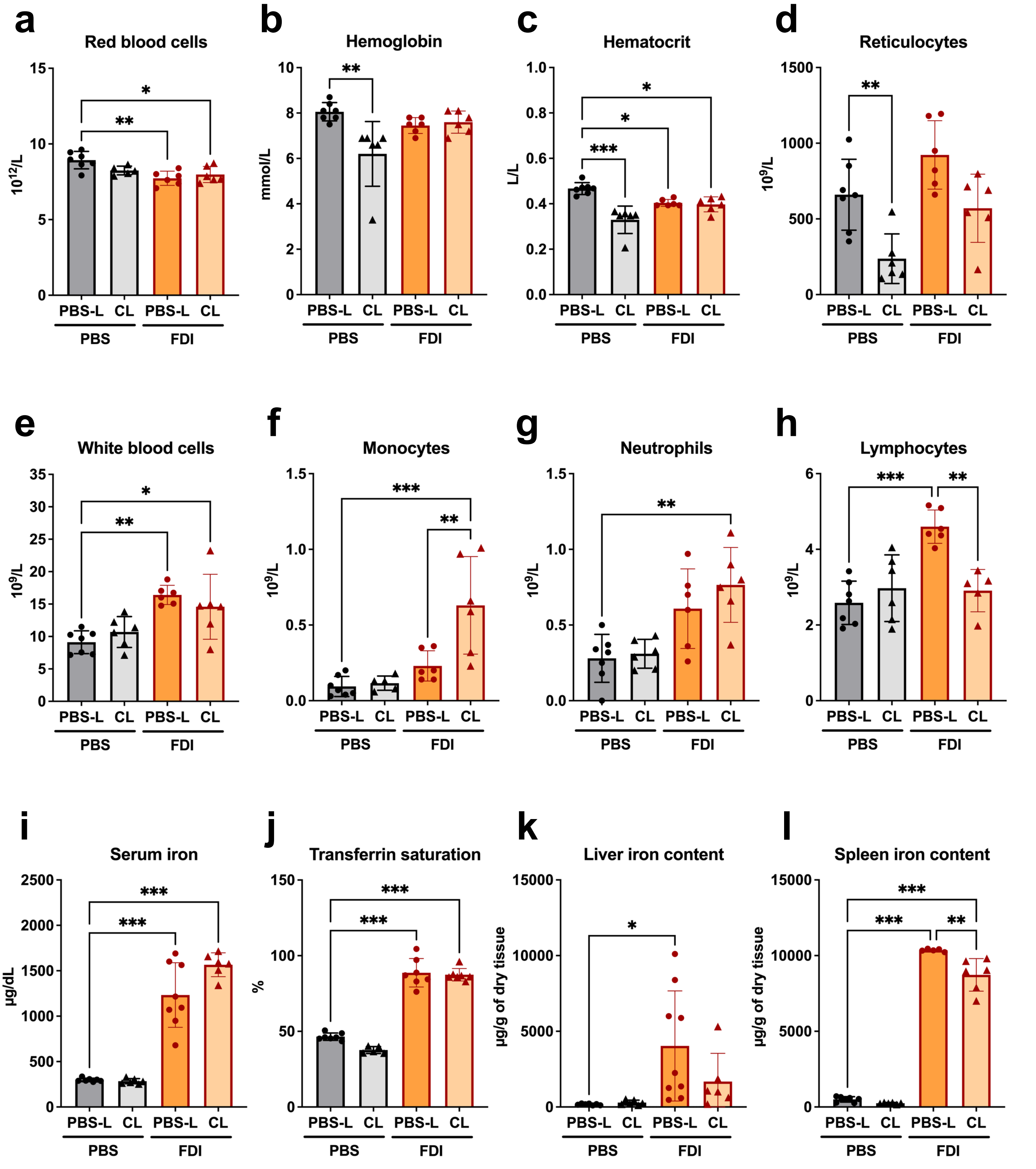

Fig. S2 Blood and iron parameters of control (PBS-L) and clodronate-treated (CL) C57BL/6J mice after receiving injections of ferric derisomaltose (FDI) for 4 weeks. Data are represented as mean ± SD (n=6-7 per group). Each symbol represents an individual animal. Statistics were calculated using a two-way ANOVA (post-hoc Tukey). *p<0.05, **p<0.01, ***p<0.001.
